# Ecophysiological Aspects and *sxt* Genes Expression Underlying Induced Chemical Defense in STX-Producing *Raphidiopsis raciborskii* (Cyanobacteria) against the Zooplankter *Daphnia gessneri*

**DOI:** 10.3390/toxins13060406

**Published:** 2021-06-08

**Authors:** Mauro C. P. Vilar, Thiago F. C. P. Rodrigues, Luan O. Silva, Ana Beatriz F. Pacheco, Aloysio S. Ferrão-Filho, Sandra M. F. O. Azevedo

**Affiliations:** 1 Laboratory Ecophysiology and Toxicology of Cyanobacteria, Carlos Chagas Filho Institute of Biophysics, Federal University of Rio de Janeiro, Rio de Janeiro 21949-902, Brazil; thiagofcpr@gmail.com (T.F.C.P.R.); luan19oliveira233@gmail.com (L.O.S.); sazevedo@biof.ufrj.br (S.M.F.O.A.); 2 Laboratory Biological Physics, Carlos Chagas Filho Institute of Biophysics, Federal University of Rio de Janeiro, Rio de Janeiro 21949-902, Brazil; biafp@biof.ufrj.br; 3 Laboratory of Evaluation and Promotion of Environmental Health, Instituto Oswaldo Cruz, FIOCRUZ, Rio de Janeiro 21040-360, Brazil; aloysio@ioc.fiocruz.br

**Keywords:** infochemicals, cyanotoxins, phytoplankton, saxitoxins, phenotypic plasticity

## Abstract

Cyanobacteria stand out among phytoplankton when they form massive blooms and produce toxins. Because cyanotoxin genes date to the origin of metazoans, the hypothesis that cyanotoxins function as a defense against herbivory is still debated. Although their primary cellular function might vary, these metabolites could have evolved as an anti-predator response. Here we evaluated the physiological and molecular responses of a saxitoxin-producing *Raphidiopsis raciborskii* to infochemicals released by the grazer *Daphnia gessneri*. Induced chemical defenses were evidenced in *R. raciborskii* as a significant increase in the transcription level of *sxt* genes, followed by an increase in saxitoxin content when exposed to predator cues. Moreover, cyanobacterial growth decreased, and no significant effects on photosynthesis or morphology were observed. Overall, the induced defense response was accompanied by a trade-off between toxin production and growth. These results shed light on the mechanisms underlying zooplankton–cyanobacteria interactions in aquatic food webs. The widespread occurrence of the cyanobacterium *R. raciborskii* in freshwater bodies has been attributed to its phenotypic plasticity. Assessing the potential of this species to thrive over interaction filters such as zooplankton grazing pressure can enhance our understanding of its adaptive success.

## 1. Introduction

Predator–prey interactions foster the evolution of defensive adaptations [1]. A series of traits that deter predation by zooplankton have been described in phytoplankton, including the formation of colonies and spines, and/or the production of harmful metabolites [2,3]. In addition to the direct effect provided by active foraging, zooplankton also release infochemicals that induce functional responses in phytoplankton species, such as increased toxin production [4,5,6,7] and colony formation or trichome stretching, which in turn may limit grazing pressure [8,9,10,11]. These infochemicals are also referred to as kairomones or predator cues, which are chemical signals released by predators that indicate a threat and can benefit prey defense. In *Daphnia*, these chemicals were found to be aliphatic sulfates and sulfamates [12,13].

In many eutrophic water bodies, cyanobacteria dominate phytoplankton and represent one of the main sources of energy for omnivorous zooplankton in aquatic food webs. Several cyanobacterial species produce bioactive metabolites regarded as anti-grazer substances because of their effects on zooplankton survivorship and reproduction [14,15,16,17], swimming and foraging behavior [18,19]. Ultimately, these metabolites can limit the role that zooplankton play in the top-down control of phytoplankton and therefore impair energy flux in aquatic ecosystems.

Because of the potential on producing these secondary metabolites also named cyanotoxins, Cyanobacteria may form nuisance blooms [20]. Microcystins (MCs), saxitoxins (STXs), cylindrospermopsins (CYNs) and anatoxins (ATXs) are among the most reported cyanotoxins. Although the chemical and pharmacological properties and biosynthesis pathways of these toxins have been well documented [21], their roles in cyanobacterial ecology remain unclear [22,23,24].

*Raphidiopsis raciborskii* (formerly *Cylindrospermopsis raciborskii* [25]) is one of the most common bloom-forming species in freshwater. It is a diazotrophic toxic cyanobacterium that was first reported in tropical and subtropical regions where it dominates (or codominates) phytoplanktonic communities. Nowadays, *R. raciborskii* has been frequently recorded in temperate freshwater environments [26]. This recent expansion and invasive (or opportunistic) potential are ascribed to its phenotypic plasticity, involving, among other traits, tolerance to a wide range of temperatures, competitive potential, and grazing resistance stemming from its morphological and metabolic versatility [26,27,28].

Some strains of *R. raciborskii* can produce toxic alkaloids with neurotoxic (saxitoxins (STXs)) or cytotoxic activity (cylindrospermopsins (CYNs)). In South America, toxic strains have been reported to be STX producers [29,30,31,32,33], while no strain has been characterized to produce CYN, although some studies have detected this toxin in environmental samples [34]. Moreover, non-toxic *R. raciborskii* strains, which usually lack genes encoding STXs or CYN [35], can also be found in the environment, co-occurring with toxic strains.

STXs are guanidinium-containing neurotoxic alkaloids that block ionic Na^+^ and Ca^2+^ channels encompassing more than 50 analogs, including non-sulfated (saxitoxin and neosaxitoxin), monosulfated (gonyautoxins), disulfated (C-toxins), and decarbamoyl variants and derivatives [36]. These toxins are distinguished from other cyanotoxins because they are produced by organisms belonging to two life domains: Bacteria (Cyanobacteria) and Eukaryota (marine dinoflagellates). In the latter, these toxins are referred to as paralytic shellfish toxins [37]. Because STX analogs display different toxicities in animal cells depending on their functional groups, toxicity equivalent factors (TEFs) are usually addressed [38].

The biosynthesis of STXs depends on enzymes encoded in a gene cluster called *sxt*, which was first described in *R. raciborskii* T3 [39], the same strain used in the present study. The *sxt* cluster comprises seven *sxt* genes, *sxtA*–*sxtD*, *sxtG*, *sxtS* and *sxtU*, which encode enzymes involved in the formation of the structural skeleton and other accessory *sxt* genes, encoding tailoring enzymes involved in chemical modifications, transport and regulation, and transposases [37].

STX-producing *R. raciborskii* have been shown to have a variety of harmful effects on zooplankton organisms, especially on daphnids, such as decreased feeding rate [40,41], mobility [17,42], growth, and reproduction [43,44]. These effects can occur via direct contact with cyanotoxins through cell ingestion or handling impairment stemming from a mismatch in the size of trichomes. On the other hand, until now no studies have examined the early responses of STX-producing cyanobacteria to chemical signals released by grazers.

The evolution of inducible defenses appears to be favored over constitutive defenses because grazing pressure by zooplankton varies on both temporal and spatial scales [45]. Defenses induced by the presence or action of predators have lower costs compared with constitutive defenses [46]. If predation can be deterred by STXs, the optimal defense hypothesis (ODH) predicts that toxin cell content should increase in response to threat cues, to maintain population fitness [7]. According to Pavia et al. [47], the ODH assumes that both chemical and morphological defenses are costly; therefore, natural selection will act to optimize their benefit–cost ratio. This leads to the prediction that there should be a trade-off with energy allocation to chemical vs. morphological defenses.

The molecular basis of grazer-induced defenses in phytoplankton is poorly understood. A study of the expression of target genes can provide insight into defensive phenotypes, and aid exploration of the adaptive traits mediating anti-predator responses. The conservation of *sxt* gene clusters in different cyanobacterial species indicates that STXs may play an adaptive role in STX-producing cyanobacteria [48]. No study to date has characterized changes in the expression of genes related to STX synthesis upon the exposure of cyanobacteria to zooplankton, nor in the more general context of predator-induced defenses. Here, we aimed to assess the effects of chemical cues released by the neotropical daphnid *Daphnia gessneri* on morphological and ecophysiological traits and *sxt* gene expression in the *R. raciborskii* strain T3. We tested the hypothesis that predator infochemicals promote a defense response (at the molecular and physiological levels) in the cyanobacterium but at an associated cost, as assumed by the ODH (see [49,50]).

## 2. Results

### 2.1. Predator-Induced Chemical Defense

**Figure 1:**
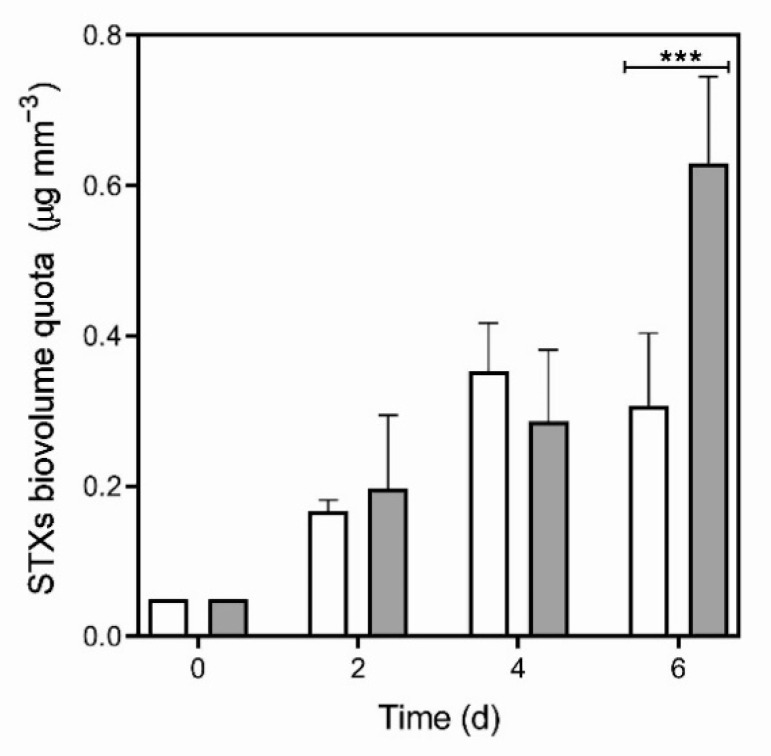
Total saxitoxin quota by biovolume of *Raphidiopsis raciborskii* T3 exposed to *Daphnia gessneri* infochemicals (gray bars) and under control conditions (white bars). Significant differences (***) = Bonferroni’s test, *p* < 0.001.

**Figure 2:**
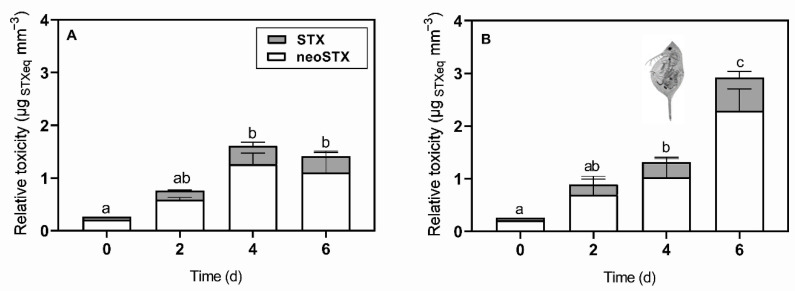
Relative biovolume toxicity based on the neosaxitoxin (white bars) and saxitoxin (gray bars) content per biovolume of *Raphidiopsis raciborskii* T3 cells (**A**) under control conditions and (**B**) exposed to *Daphnia gessneri* infochemicals. Different letters indicate significant differences (Bonferroni’s test, *p* < 0.05).

*D. gessneri* infochemicals significantly enhanced the total STXs production in *R. raciborskii* T3 over time (Figure 1; Supplementary Table S1). On the 6th day of incubation with infochemicals, the cellular amount of STXs expressed as STX biovolume quota was 0.63 ± 0.12 µg mm^−3^. This amount was twice that measured in the control (0.31 ± 0.10 µg mm^−3^; Bonferroni’s test, *p* < 0.001), which indicated that a chemically induced defense response was displayed by the cyanobacterium (Figure 1). In addition, neoSTX and STX content increased significantly in response to the infochemicals, which resulted in a higher cellular relative toxicity (infochemicals = 2.92 ± 0.53 µg STX_eq_ mm^−3^; control = 1.41 ± 0.47 µg STX_eq_ mm^−3^; Bonferroni’s test, *p* < 0.001) (Figure 2; Supplementary Table S2).

**Figure 3:**
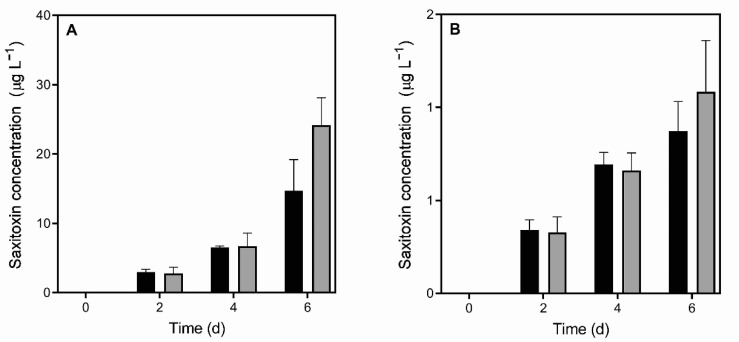
Concentrations of (**A**) intracellular and (**B**) extracellular saxitoxin in *Raphidiopsis raciborskii* T3 under control conditions (black bars) and exposed to *Daphnia gessneri* infochemicals (gray bars). No significant differences were detected between the two conditions.

There was no significant effect of kairomones on the total pool of dissolved STXs (Figure 3). Predator cues resulted in an STX release rate of 0.20 ± 0.04 µg L^−1^ d^−1^, while under control conditions, the release rate was 0.16 ± 0.03 µg L^−1^ d^−1^ (data not shown).

Overall, the STX biovolume content, relative biovolume toxicity and volumetric STX concentration increased over time (two-way RM ANOVA, *p* < 0.01) (Figure 1, Figure 2 and Figure 3). The latter also reflected the increase in cell concentration in both experimental conditions.

**Figure 4:**
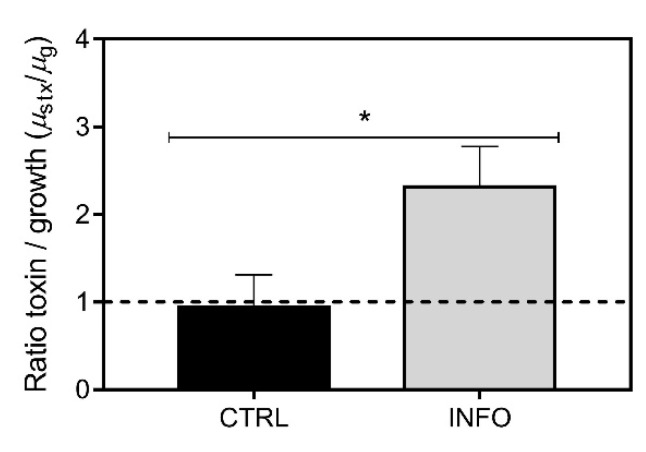
Ratio between specific total saxitoxin production rate (µ_stx_) and specific growth rate (µ_g_) obtained from first-order rate kinetics (log phase) for *Raphidiopsis raciborskii* T3 grown under control conditions (CTRL) and with *Daphnia gessneri* infochemicals (INFO). The dashed line indicates a 1:1 relationship between µ_stx_ and µ_g_. (*) = *t*-test, *p* < 0.05.

First-order rate kinetics showed different patterns in toxin production/growth in *R. raciborskii* T3. Under control conditions, STX production was coupled to growth (1:1 μ_stx_/μ_g_ ratio) (Figure 4). In contrast, predator cues caused the mean value of the μ_stx_/μ_g_ ratio to be significantly greater than 1 (Student’s *t*-test; T = 4.055, *p* < 0.05), indicating that the specific rate of STX production was faster than the specific growth rate, resulting in increased cell toxin accumulation (Figure 1).

### 2.2. STX Gene Expression

**Figure 5:**
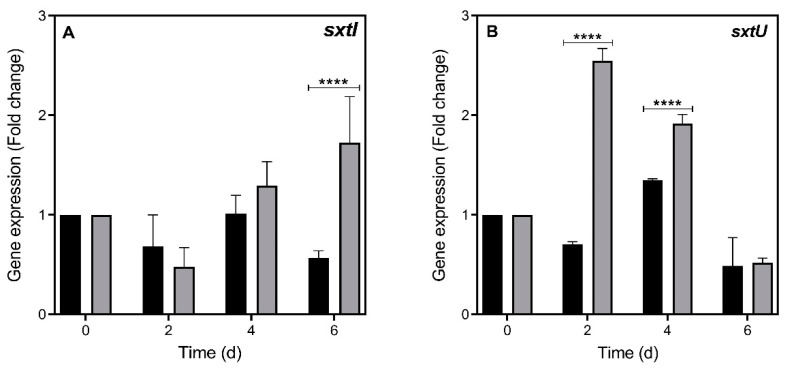
Relative expression of (**A**) *sxtI* and (**B**) *sxtU* genes in *Raphidiopsis raciborskii* T3 under control conditions (black columns) and exposure to *Daphnia gessneri* infochemicals (gray columns). Fold change calculated by the 2^−ΔΔCT^ method. Significant differences between the two conditions are indicated by asterisks (****) = Bonferroni’s test, *p* < 0.0001.

Significant up-regulation of the expression of two STX-related genes, *sxtI* (two-way RM ANOVA, F_(1,4)_ = 8.588; *p* < 0.05) and *sxtU* (two-way RM ANOVA, F_(1,4)_ = 153.2; *p* < 0.001), was detected in *R. raciborskii* T3 grown in the presence of infochemicals compared with the control (Figure 5; Supplementary Table S3). In the presence of *Daphnia* infochemicals, *sxtI* expression was significantly higher than the control at the 6th day of incubation (Bonferroni’s test, *p* < 0.0001) (Figure 5A), which was also when a significant increase in STX production was observed (see Figure 1). Transcript levels increased over time (F_(3,12)_ = 8.172; *p* < 0.01), and a significant interaction between both factors (time × treatment, F_(3,12)_ = 10.13; *p* < 0.01) was observed. Transcript abundances of *sxtU* were higher in the treatment than in the control on the 2nd and 4th days (F_(1,4)_ = 153.2; *p* < 0.001) (Bonferroni’s test, *p* < 0.0001) (Figure 5B), four days before an increase in STX content was detected. There was also a significant interaction in the transcript levels of this gene between both factors (time × treatment, F_(3,12)_ = 85.65; *p* < 0.0001). The abundance of *sxtU* transcripts decreased gradually over time, and on the 6th day, it was similar under both treatment and control conditions (F_(3,12)_ = 137.7; *p* < 0.0001).

### 2.3. Growth, Morphology and Photosynthetic Parameters

**Figure 6:**
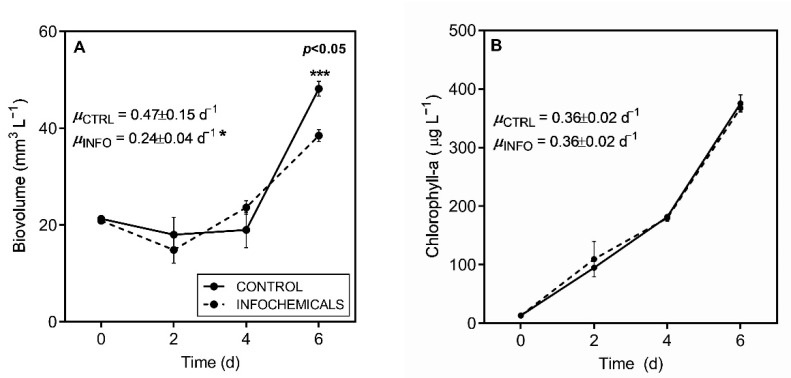
Growth curves and growth rates estimated by (**A**) biovolume and (**B**) chlorophyll-a concentration for *Raphidiopsis raciborskii* T3 exposed to *Daphnia gessneri* infochemicals (dashed line) and under control conditions (solid line). Significant differences (*) = *p* < 0.05; (***) = *p* < 0.001, Bonferroni’s test.

*R. raciborskii* T3 displayed a significant decrease in biovolume (two-way RM ANOVA, *p* < 0.001) and specific growth rate when exposed to *Daphnia*-conditioned medium compared with the control (µ_control_ = 0.47 ± 0.15 day^−1^; µ_infochemicals_ = 0.24 ± 0.04 day^−1^; Student’s *t*-test, *p* < 0.05) (Figure 6A; Supplementary Table S4). No differences were observed in chlorophyll-a concentrations (Figure 6B).

Additionally, growth in the presence of predator infochemicals did not affect photosynthesis, as estimated by the light-harvesting efficiency (α), relative PSII quantum yield (Fv’/Fm’), light saturation parameter (*Ik*) and maximum electron transport rate (ETR_max_) (Table 1). However, a significant increase in the photosynthetic parameters ETR_max_ (two-way RM ANOVA, *p* < 0.05) and *Ik* (two-way RM ANOVA, *p* < 0.001) over time was observed (Table 1).

**Figure 7:**
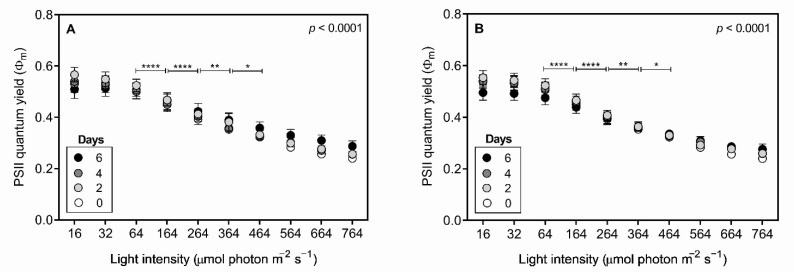
Maximum PSII quantum yield derived from the photosynthesis irradiation curve of *Raphidiopsis raciborskii* T3 control (**A**) and exposed (**B**) to *Daphnia gessneri* infochemicals for 6 days. Significant differences (* *p* < 0.05; ** *p* < 0.01; **** *p* < 0.0001) after Bonferroni’s test.

The irradiation curves of *R. raciborskii* T3 (maximum PSII quantum yield) indicated no effect of predator infochemicals (Figure 7). Under both control and treatment conditions, the cells were sensitive to light intensities above 64 µmol photons m^−2^ s^−1^, and *R. raciborskii* T3 displayed a significant decrease in photosynthetic efficiency when exposed to increased light pulses (two-way RM ANOVA, *p* < 0.0001) (Figure 7).

The morphology of *R. raciborskii* T3 did not change (Dunnett’s test, *p* > 0.05) when exposed to infochemicals. Trichomes ranged from an initial length of 74.91 ± 32.54 µm to 75.05 ± 40.44 µm at day 6 in the presence of *Daphnia* infochemicals, and from 74.91 ± 32.54 µm to 79.69 ± 42.79 µm under control conditions. Similarly, no changes were detected in trichome thickness, with mean widths of 2.63 ± 0.52 µm and 2.53 ± 0.43 µm under control and infochemical conditions, respectively.

## 3. Discussion

Infochemicals released by *D. gessneri* significantly reduced growth but enhanced *sxt* gene expression and toxin production in *R. raciborskii* T3, evidencing a predator-induced chemical defense. We observed these effects using a density of cladocerans near to those naturally occurring, and we detected an approximately two-fold increase in *sxt* gene transcript levels and cellular STX quotas of the cyanobacterium in response to zooplankton alarm cues.

Several studies have characterized the physiological responses underlying predator-induced chemical defenses in microcystin-producing cyanobacteria [5,51,52,53], including gene expression [45,54]. In contrast, an increase in STX production mediated by predator cues has only been reported in eukaryotic marine dinoflagellates. For example, Selander et al. [7] found that there was a more than two-fold increase in STX production by *Alexandrium minutum* when exposed to kairomones of the copepod *Acartia tonsa*. Similar findings were obtained for STX-producing *A. tamarense* and *A. fundyense* exposed to different predators [55,56,57]. Despite the effect that the C:N:P ratio has on STX production as suggested by the stoichiometric hypothesis [58], the cellular content of STX can increase in response to predator cues, regardless of nutrient conditions [59].

*R. raciborskii* T3 has *sxtM*/*sxtF* genes that encode multidrug and toxic compound extrusion (MATE) transport proteins [60]. However, no effect on STX secretion by this cyanobacterium was observed, despite the increased biovolume toxin content it displayed following exposure to *Daphnia* infochemicals. In fact, increasing intracellular toxin content is an effective cyanobacterial defense against grazing pressure by zooplankton as opposed to simply releasing these metabolites into the surroundings. Feeding is thought to be the most important route of exposure to toxins in aquatic systems [61]. This assumption is based on the harmful acute effects and life-history impairments of zooplankton, observed when they actively feed on STX-producing cyanobacteria [16,17,19,62,63].

According to Yang et al. [64], the kairomone-induced increase in STX production is mediated by chemoreception and does not involve mechanical damage. This defensive response does not depend on active grazing and must be assessed by exposing harmful algae or cyanobacteria to predator kairomones. Experiments involving direct exposure would lead to selective grazing on less toxic cells, which might favor cells with a higher mean toxin content; these cells may be indistinguishable from those in which toxin production had been induced [7].

In our study, two representative genes of the *sxt* cluster, *sxtI* and *sxtU*, showed increased transcript levels in *R. raciborskii* cells exposed to *D. gessneri* infochemicals. This response preceded the observed increase in STX cellular quotas. *sxtU* encodes a dehydrogenase that reduces the terminal aldehyde group of the STX precursor, whereas *sxtI* encodes a carbamoyl transferase that catalyzes carbamoyl transfer from carbamoyl phosphate onto the free hydroxyl at C-13, forming STX [60]. These genes are reliable markers for assessing STX synthesis because they encode enzymes that participate in key steps of STX formation. Other studies have successfully used these *sxt* molecular markers as reporters to assess STX biosynthesis in *R. raciborskii* [65,66]. Yang et al. [64] were the first to demonstrate an increased cellular toxin content and associated transcript profile of a STX-producing dinoflagellate exposed to zooplankton cues, although no genes of the *sxt* cluster were identified. Later, Wohlrab et al. [67] assessed *sxtA* gene expression coupled with STX production in *A. fundyense* exposed to predators. Despite a two-fold increase in the STX cell quota, no changes were observed at the transcript level. The authors suggested that *sxtA* might have been overexpressed early at the onset of the dinoflagellate defense response prior to the observed physiological changes. This assumption is confirmed in our findings, where we demonstrated that an increase in *sxt* transcript levels preceded the increase in the STX cellular quota during the cyanobacterial response to predator cues.

Molecular events involved in induced chemical defenses in cyanobacteria are still poorly explored, especially in STX-producing species. Although the presence of genes related to toxin synthesis precedes the existence of metazoans [68], these toxins might have evolved to diversify their functions and represent an adaptive response against predators [23]. This is exemplified by the predator-mediated induction of STX production coupled to the increased resistance to grazers in *A. minutum* [7]. Thus, it is likely that the potential adverse effects of STXs on zooplankton have undergone positive selection more recently, as has been suggested for MCs by Rzymski et al. [69].

In addition to the increased toxin production induced in response to predators, the growth of *R. raciborskii* T3 decreased, and biovolume concentrations were low under these conditions. Overall, the imbalance in growth and STX production observed after exposure to zooplankton infochemicals indicates the presence of a trade-off. According to photosynthetic parameters, *R. raciborskii* cells were in a normal physiological state, but growth was still reduced. It is likely that the potential threat indicated by the infochemicals caused the cells to allocate energy toward toxin production instead of cell division. Indeed, studies examining the effects of environmental stressors on *R. raciborskii* physiology have suggested that there is a trade-off between growth and toxin production [33]. Different stress conditions can activate similar physiological responses as cyanotoxin production. Studies with both STX- and CYN-producing *R. raciborskii* strains have demonstrated this imbalance between growth and toxin production in response to abiotic stress (e.g., nutrient limitation, light and temperature stress) [30,70,71,72]. However, our study is the first to report the response of the cyanobacterium *R. raciborskii* to biotic stress caused by predation. Additionally, costs are not only related to the production of well-described toxins, but also to other unknown metabolites. For example, Blossom et al. [73] studied the costs of toxicity in STX-producing *Alexandrium* and did not find any growth reduction related to toxin production; instead, growth reduction was related to lytic activity, which was associated with other poorly known metabolites.

Additionally, a trade-off response was also confirmed through the first-order kinetics rate. The calculated ratio between the rates of toxin production and cell division indicated that induced chemical defense in *R. raciborskii* T3 led to a greater increase in the STX production rate than in growth. The production of other cyanotoxins, such as microcystins, is considered a constitutive process that is directly coupled to cell division [74,75]. Cells can achieve an up to three-fold variation in the toxin quota depending on their growth stage [76]. However, the cellular STX content depends not only on the cell division rate but also on environmental factors [33].

Generally, photosynthesis is thought to be disrupted when the cellular growth of phytoplankton is reduced, as the photosynthetic machinery is key for biomass acquisition. In our study, although infochemicals have negatively affected *R. raciborskii* T3 growth, no photosynthetic impairments were observed. However, a significant increase in the ETR_max_ and *Ik* parameters over incubation time occurred, indicating enhanced light acquisition by *R. raciborskii*, which suggests that self-shading was caused by the accumulation of cells.

We examined photosynthetic parameters to assess early changes in cyanobacterial physiology, as little is known about how predator-induced defenses in phytoplankton might affect photosynthesis. Savic et al. [77] evaluated the photosynthetic response of toxic and nontoxic *Microcystis* strains exposed to *D. magna* kairomones. Decreases in photosynthetic activity and chlorophyll-a content in the toxic strain were observed in response to *Daphnia*. Savic et al. [78] reported that zooplankton cues did not affect *M. aeruginosa* photosynthetic activity under a similar experimental set-up, which was probably explained by the fact that only ETR_max_ was examined. In our study, the finding that zooplankton infochemicals had no effect on photosynthesis was based on a more comprehensive set of photosynthetic parameters (i.e., *Ik*, ETR_max_, α and ϕ_m_), in addition to the commonly used chlorophyll-a concentration, ETR and PSII quantum yield.

We also characterized trichome length and thickness, which are proxies of induced morphological defense; however, no significant changes were observed. Several studies have examined the effects of predator kairomones on cyanobacterial filament morphology, and most studies have reported an increase in width [11,79]; however, less is known about the effect on trichome length [11,80]. Filamentous morphology acts as a functional trait that prevents ingestion by clogging the predator filtration apparatus, and some microalgae can adjust their length in response to predator cues to minimize losses associated with zooplankton grazing pressure [81,82,83,84].

Defenses induced under variable grazing threat/attack are considered more energetically efficient than constitutive ones because they enable organisms to maximize benefits and reduce the costs associated with the investment in the constant expression of defensive strategies [85,86]. Numerous studies have attempted to estimate the costs and benefits of defense mechanisms in phytoplankton; Pančić and Kiørboe [87] argue that the costs associated with induced defenses have largely been undetermined. They also assume that if there is no cost to predator-resistance trait expression, all species would evolve toward a state of equal defense, and the community would not be structured by predation. This claim is consistent with the ODH, which is based on the fundamental assumption that the benefits of defense outweigh the costs, especially under high predation pressure [49]. Although the ODH was originally proposed for plant defenses, this hypothesis also predicts defensive responses in unicellular organisms, including phytoplankton, where phenotypically plastic organisms can express defensive traits upon exposure to a threat. However, intraspecific variability in these responses indicates that the associated costs are genotype-specific [53]. Thus, the adaptive evolution of defensive traits can be mediated by both Mendelian (e.g., genotype-specific responses) and non-Mendelian (e.g., phenotypic plasticity) evolving mechanisms.

In general, the predominance of *R. raciborskii* in water bodies has been attributed to its phenotypic plasticity and its dispersal capacity [26,27]. Assessing the ability of this species to cope with diverse ecological interaction filters (e.g., competition, predation and parasitism) [88] could provide insight into the complex factors that contribute to its adaptive success. Our results point out the potential of a STX-producing strain of the invasive (opportunistic) cyanobacterial species *Raphidiopsis raciborskii* in responding to grazer cues with an increase in toxin production at different levels. As cyanotoxins are also regarded as anti-grazing chemicals, describing an induced chemical defense and other underlying responses in this species adds more information into the knowledge of adaptive traits (e.g., grazing resistance) which allow its wide occurrence and dominance in several lakes. Additionally, regarding the ecological relevance of our experimental model, *R. raciborskii* has been reported co-occurring with *D. gessneri*, in addition to other *Daphnia* species, in several water bodies [89,90,91].

In conclusion, *D. gessneri* infochemicals induced the up-regulation of *sxt* genes and led to a subsequent increase in toxin content. On the other hand, *R. raciborskii* reduced its growth. These results support the hypothesis that the anti-predator defenses of *R. raciborskii* may be costly.

## 4. Materials and Methods

### 4.1. Phytoplankton and Zooplankton Cultures

*R. raciborskii* T3 is an STX-producing strain isolated from Billings Reservoir (São Paulo, Brazil) [29]. In this cyanobacterial strain, neoSTX and STX analogs are predominantly produced [92]. Stock cultures were maintained in ASM-1 medium [93] at 24 ± 1 °C and 50 µmol photons m^−2^ s^−1^ under a 12/12-h light-dark cycle. Under these conditions, T3 had an average trichome length of 74.91 ± 32.54 µm.

Zooplankton consisted of the neotropical species *Daphnia gessneri* isolated from Barra do Braúna Reservoir (Minas Gerais, Brazil) in 2018. Individuals were kept in RT medium [94] enriched with commercial 0.1% (~2.25 mg C L^−1^) humic extract (Microbe-lift^®^ Amazon Black & Soft Water Conditioner, USA) at an initial pH 7.6, 24 ± 1 °C, 50 µmol photons m^−2^ s^−1^ and a 12/12-hour dark-light cycle. Animals were fed a cell suspension of the green algae *Selenastrum capricornutum* at a final concentration of 500 µg C L^−1^ once every two days. The algae population was dominated by unicells (mean length and width dimensions: 13.80 ± 1.56 × 3.18 ± 0.66 µm).

### 4.2. Experimental Set-Up

#### 4.2.1. Zooplankton Filtrate to Obtain Infochemicals

Prior to experiments, *D. gessneri* clones were starved in fresh RT medium to empty their guts and remove any superficial contamination. Cultures were established with adults at a populational density of 60 ind L^−1^. The animals were fed with a cell suspension of the green algae *S. capricornutum* at 500 µg C L^−1^ and incubated for 96 h. Thereafter, animals were removed using a cup of plankton (60-µm mesh size), and the zooplankton medium was sterilized by filtration through a 0.22-µm pore membrane. The filtrate (zooplankton pre-conditioned medium) was used to prepare the ASM-1 culture medium, hereafter referred to as infochemicals. As a control, RT medium with the chlorophycean as food suspension at a similar concentration provided for the treatment with *Daphnia* wasalso incubated for 96 h, and the medium was then filtered through a 0.22-µm pore membrane. The filtrate was also used instead of water to prepare ASM-1 and transferred together with the infochemical-rich medium to the predator-induced defense experiment.

#### 4.2.2. Predator-Induced Defenses Experiment

Zooplankton-conditioned medium was used to evaluate whether *R. raciborskii* cells respond to predator infochemicals. Biomass inocula of *R. raciborskii* T3 at 20 mm^3^ L^−1^ (10^5^ cells mL^−1^) were prepared in 1-L Erlenmeyer flasks filled with 600 mL of infochemical-rich or control culture medium (*n* = 3). Batch cultures were incubated over 6 days at 24 ± 1 °C and 50 µmol photons m^−2^ s^−1^ under a 12/12-h dark-light cycle. Samples were collected every two days to evaluate growth, morphological changes, photosynthetic activity, toxin content and the expression of *sxt* genes.

### 4.3. Growth, Morphology and Photosynthetic Measurements

Samples were harvested and preserved with 1% acetic Lugol’s solution for trichome count in a Fuchs-Rosenthal chamber under an optical microscope (Olympus BX51). The results are expressed in trichomes mL^−1^. Trichome density was converted to biovolume (mm^3^ L^−1^) based on the mean trichome volume (µm^3^) [95,96].

The specific growth rate was calculated during the exponential growth phase using Equation (1):

(1) $$µ=\frac{lnN_{2}-lnN_{1}}{t_{2}-t_{1}}$$

where *N*_2_ = final biovolume; *N*_1_ = initial biovolume; *t*_2_ = final time; and *t*_1_ = initial time.

Additionally, trichome length and width (µm) at *t*_initial_ and *t*_final_ in both culture conditions were measured to examine morphological variation. Samples were analyzed under an Olympus BX51 light microscope (250× and 400× magnification) coupled to an image-capture system (Canon t6i). Trichomes were measured using the Cell^B software package for image acquisition.

Photosynthetic activity was evaluated using a PHYTO-PAM fluorometer (Heinz Walz GmbH, Germany) equipped with a PHYTO-EDF detection unit for measuring cyanobacterial fluorescence. Saturation pulses (36 µmol photons m^−2^/s^−1^) were applied, and fluorescence data were converted to chlorophyll-a concentration (μg L^−1^) and photosynthetic yield (relative Fv’/Fm’). Light curves were performed with dark-acclimated culture samples (F_0_) with 10-s intervals between pulses. Light intensity varied from 16 to 764 µmol photons m^−2^/s^−1^. From these curves, the values of the maximum electron transport rate (ETR_max_), light-harvesting efficiency (α) and light saturation parameter (*Ik*) were obtained as a function of irradiance. Additionally, the maximum effective quantum efficiency of PSII was calculated as ϕ_m_ = [(Fm − F)/Fm], where F is the fluorescence of the dark-adapted sample and Fm is the maximum dark-adapted fluorescence.

### 4.4. STX Analyses and Toxicity Equivalency Calculation

Aliquots were taken from *R. raciborskii* cultures, and cells were harvested by centrifugation (7200× *g*, 10 min, 4 °C). The supernatant was filtered (0.45-μm pore membrane) for toxin measurements in the dissolved fraction, and the pellet was used to measure the intracellular toxin content. Both samples were frozen at –20 °C, freeze-dried and then subjected to STX extraction. For cellular toxin extraction, 0.5 M acetic acid solution was added to the dried cells in 3 cycles of 1 h each. At each cycle, the cell extract was centrifuged as previously mentioned, and the supernatant was reserved. For dissolved STX recovery, filtered samples were freeze-dried and then resuspended in a 0.5 M acetic acid solution. Both dissolved and cellular fractions were filtered in a 0.22-µm syringe filter and stored in 1.5-mL vials prior to chromatographic analysis.

The analysis was performed on a Shimadzu Class VP liquid chromatography system with a fluorescence detector (RF-10A XL). A reversed-phase C18 column (Lichrospher^®^; 150 mm × 4.6 mm; 5 µm-Merck) and a 20-µL loop injector were used. Chromatographic analyses were performed according to the post-derivatization method [97] using a mobile phase of 2 mM sodium 1-heptane sulfonate in 30 mM ammonium phosphate and 5% acetonitrile at a flow rate of 0.8 mL min^−1^. STXs were detected at an excitation wavelength of 330 nm and an emission wavelength of 400 nm.

For quantification of toxins, standard solutions of STX and neoSTX from the National Research Council (NRC)—Institute of Marine Biosciences (Canada) were used. Quantified STXs were expressed as volumetric intra- and extracellular concentrations (µg L^−1^), and cellular toxin amounts as STXs quota per biovolume (µg mm^−3^).

Total relative cellular toxicity (saxitoxin equivalents, STX_eq_) was expressed as biovolume toxicity µg STX_eq_ mm^−3^) according to the TEF described for each STX analog (in this study, neoSTX and STX) in FAO/WHO [98] and using Equation (2):

(2) $$STXeq=\overset{n}{\underset{i=1}{\sum }}C_{i}\times TEF_{i}$$

where *C_i_* is the concentration of the individual toxin analog and its assigned *TEF_i_*.

### 4.5. Application of First-Order Rate Kinetics to Assess Total STX Production

The specific growth rate (μ_g_) and the specific total STX production rate (μ_stx_) were calculated during the exponential growth phase according to simple first-order rate kinetics using either biovolume concentration (mm^3^ L^−1^) or volumetric total intracellular STX data (μg L^−1^), respectively. Both specific rates are reported in units per day (d^-l^).

The ratio between μ_stx_ and μ_g_ was calculated to assess different patterns of toxin production coupled to the growth cycle and subsequent changes in the toxin cell (or biovolume) quota (*Q*_tox_) as described in Orr et al. [74,75], such that:

Equation (3) describes the condition where the intracellular μ_stx_ between *t*_0_ and *t*_n_ is slower than μ_g_, resulting in a lower *Q*_tox_.

(3) $$0.5<\frac{{µ}_{\mathrm{stx}}}{{µ}_{g}}<1$$

Equation (4) describes the condition where the intracellular μ_stx_ between *t*_0_ and *t*_n_ is a function of μ_g_, resulting in a constant *Q*_tox_ (1:1 growth-toxin relationship).

(4) $$\frac{{µ}_{\mathrm{stx}}}{{µ}_{g}}=1$$

Equation (5) describes the condition where the intracellular μ_stx_ between *t*_0_ and *t*_n_ is higher than μ_g_, resulting in an increased *Q*_tox_.

(5) $$\frac{{µ}_{\mathrm{stx}}}{{µ}_{g}}>1$$

### 4.6. Expression of Genes Involved in STX Biosynthesis

Transcript levels of the *sxtI* and *sxtU* genes were measured. Although most steps in the biosynthesis pathway of STX are putative, sequence similarity analysis indicates that the *sxtU* gene encodes a dehydrogenase that reduces the terminal aldehyde group of the STX precursor [60], and the *sxtI* gene encodes a carbamoyltransferase that catalyzes carbamoyl transfer from carbamoyl phosphate onto the free hydroxyl at C-13, forming STX. We chose these genes based on previous studies that reported a coherent response pattern of *sxtI* and *sxtU* and the other genes of the *sxt* cluster, and a coherent response of *sxtI* and *sxtU* expression and STX levels [65,66]. The reference gene used to calculate the relative expression of the target genes was *rpoC1*, which encodes the RNA polymerase gamma subunit [99].

Samples of 50 mL were taken from *R. raciborskii* cultures, and cells were harvested by centrifugation (1500× *g*, 15 min, 4 °C). TRIzol^®^ (300 μL) was added to the pellets, and the suspensions were immediately stored at –80 °C. RNA extraction was performed using the Direct-zolTM RNA Miniprep Plus (Zymo Research^®^) extraction kit per the manufacturer’s instructions (including a DNAse digestion step). RNA samples were suspended in RNAse-free water, and the nucleic acid quality and concentration were determined using the RNA HS Assay kit (Life Technologies) in a Qubit fluorometer (Thermo Fisher Scientific). To check for DNA contamination, PCR was carried out using 16S rDNA primers and purified RNA samples as templates. cDNA synthesis was performed with the GoScript Reverse Transcriptase enzyme (Promega) per the manufacturer’s instructions. The cDNA products were quantified in a Nanodrop, diluted 1:10 and tested for amplification with specific primers for *rpoC1* [99].

The obtained cDNA was used in qPCR to quantify the relative abundance of *sxtU* and *sxtI* transcripts. For each sample, 3 µL of a 1:10 cDNA dilution was mixed with 7.5 μL of Sybr Green PCR MasterMix (Applied Biosystems), 0.6 µL of a 20 µM solution of each primer and nuclease-free water to a final volume of 15 µL. Reactions were performed with the following primers: *sxtU* forward [5′-ACTCCCAGAACATTCACATCG-3′]—*sxtU* reverse [5′-GGAATTGGTGTGTTTGGTGC-3′] [99]; *sxtI* forward [5′-TGCAGTGGGAGCAGCTTTAG-3′]—*sxtI* reverse [5′-GATCGCCTGCTGTTGAAGTG-3′] [66]; and *rpoC1* forward [5′-GACATGGTTTTGGGAGCCTA-3′]—*rpoC1* reverse [5′-CGTTATCCGGTTGTCCTGTT-3′] [100]. For each sample, triplicate reactions were run on a QuantStudio 3 Real-Time PCR system (Thermo Fisher Scientific), with a preincubation at 95 °C for 10 min and 40 cycles of amplification at 95 °C for 15 s and 60 °C for 1 min. A melting curve was obtained by incubation at 95 °C for 15 s, 60 °C for 1 min and 95 °C for 15 s. Negative controls for each primer set were included in which nuclease-free water was used instead of cDNA template. The amplification efficiency of each primer set was calculated from standard curves using serial dilutions of cDNA, according to the equation E = 10 (−1/slope) (Supplementary Figure S1 and Table S5). Transcript levels of the target *sxt* genes were normalized to the reference *rpoC1* gene, and the relative change in transcript levels was calculated using the ΔΔCT method of relative quantification [101].

### 4.7. Statistical Analysis

All data were checked for normality and homoscedasticity of variances. Specific growth rate data and the ratio between specific toxin production and growth rate were analyzed by Student’s *T*-test to test for significant effects of zooplankton cues. Variation in the growth, total STXs biovolume quota, biovolume relative toxicity, total STXs pool size and *sxt* gene expression over incubation time were verified using repeated measures of two-way ANOVA with a post-hoc Bonferroni’s test. Morphological trait variation was analyzed using one-way ANOVA with a post-hoc Dunnett’s test using the morphological trait at the initial time as a control condition. The ΔΔCT data were log-transformed, and two-way RM ANOVA was used to compare the infochemical-rich samples with the control samples on each day. All analyses were performed, and graphs were produced using GraphPad Prism 7.0 software.

## Figures and Tables

**Table 1:** Photosynthetic parameters of *Raphidiopsis raciborskii* T3 exposed to *Daphnia gessneri* infochemicals (INFO) and under control conditions (CTRL). Yield—relative PSII quantum yield—at a saturation pulse of 36 PAR (photosynthetically active radiation), ETR_max_—maximum electron transport rate, *Ik*—light saturation parameter, and alpha (α)—light-harvesting efficiency. SD = standard deviation. Values with the same letter are not significantly different (Bonferroni’s test, *p* < 0.05).

day	Yield		ETR_max_		Ik		Alpha	
	(Relative Fv’/Fm’)		(µmol e m^−2^ s^−1^)		(µmol photon m^−2^ s^−1^)		(µmol photon m^−2^ s^−1^)	
	CTRL ^1^	INFO ^1^	CTRL	INFO	CTRL	INFO	CTRL	INFO
**0**	0.48 ± 0.0 ^a^	0.48 ± 0.0 ^a^	81.80 ± 0.0 ^a^	81.80 ± 0.0 ^a^	353.10 ± 0.0 ^a^	353.10 ± 0.0 ^a^	0.23 ± 0.0 ^a^	0.22 ± 0.0 ^a^
**2**	0.59 ± 0.0 ^a^	0.48 ± 0.1 ^a^	104.07 ± 16.5 ^a^	77.70 ± 31.4 ^a^	429.23 ± 66.7 ^a^	359.10 ± 69.3 ^a^	0.23 ± 0.0 ^a^	0.23 ± 0.0 ^a^
**4**	0.55 ± 0.0 ^a^	0.53 ± 0.0 ^a^	103.27 ± 15.3 ^a^	102.47 ± 8.1 ^a^	448.03 ± 56.9 ^a^	440.23 ± 36.2 ^a^	0.24 ± 0.0 ^a^	0.23 ± 0.0 ^a^
**6**	0.52 ± 0.0 ^a^	0.51 ± 0.0 ^a^	118.27 ± 5.7 ^a^	109.77 ± 6.7 ^a^	531.03 ± 41.9 ^a^	514.60 ± 41.9 ^a^	0.22 ± 0.0 ^a^	0.23 ± 0.0 ^a^

^1^ All data are shown as mean ± standard deviation.

## Data Availability

Data is contained within the article or Supplementary Materials.

## Supplementary Materials

The following are available online at https://www.mdpi.com/article/10.3390/toxins13060406/s1, Table S1: Results of the two-way ANOVA for differences in total saxitoxins biovolume quota of *Raphidiopsis raciborskii* T3 exposed (infochemicals) and not exposed (control) to *Daphnia gessneri* over 6 days of incubation. Table S2: Results of the post hoc Bonferroni’s comparison test for differences in biovolume toxicity relative to STX analogues produced by *Raphidiopsis raciborskii* T3 exposed (infochemicals) and not exposed (control) to *Daphnia gessneri* over 6 days of incubation. Table S3: Results of the two-way ANOVA for differences in the relative expression of *sxtI* and *sxtU* by *Raphidiopsis raciborskii* T3 exposed (infochemicals) and not exposed (control) to *Daphnia gessneri* over 6 days of incubation. Table S4: Results of the two-way ANOVA for differences in the biovolume growth curves and chlorophyll-a of *Raphidiopsis raciborskii* T3 exposed (infochemicals) and not exposed (control) to *Daphnia gessneri* over 6 days of incubation. Table S5: Efficiencies and standard curve parameters obtained by real-time qPCR analysis for the cyanobacterial *rpoC1*-, *sxtI*- and *sxtU*-specific primer sets. Figure S1: Standard curve and its linear equation for cyanobacterial *rpoC1*-, *sxtI*- and *sxtU*-specific primers.
