# Supplementary material for: Ecophysiological Aspects and sxt Genes Expression Underlying Induced Chemical Defense in STX-Producing Raphidiopsis raciborskii (Cyanobacteria) against the Zooplankter Daphnia gessneri
Source: Toxins (Basel). 2021 Jun 8;13(6):406. doi: 10.3390/toxins13060406 (PMC8230027; doi:10.3390/toxins13060406)
Supplement: Supplementary file 1 [file toxins-13-00406-s001.zip › toxins-1233174-supplementary.pdf]

Supplementary Materials: Ecophysiological Aspects and *sxt* Genes Expression Underlying Induced Chemical Defense in STX-Producing *Raphidiopsis raciborskii* (Cyanobacteria) Against the Zooplankter *Daphnia gessneri*

Mauro C. P. Vilar, Thiago F. C. P. Rodrigues, Luan O. Silva, Ana Beatriz F. Pacheco, Aloysio S. Ferrão-Filho and Sandra M. F. O. Azevedo

Table S1. Results of the two-way ANOVA for differences in total saxitoxins biovolume quota of *Raphidiopsis raciborskii* T3 exposed (infochemicals) and not exposed (control) to *Daphnia gessneri* over 6 days of incubation. ns = no significant differences.

STXs Biovolume Quota	SS	Df	MS	*F*	*P*
Time	0.5828	3	0.1943	$F_{(3,12)} = 31.02$	<0.0001
Infochemicals	0.0308	1	0.0308	$F_{(1,4)} = 7.524$	ns
Interaction	0.134	3	0.0447	$F_{(3,12)} = 7.133$	<0.01
Subjects (matching)	0.0164	4	0.0041	$F_{(4,12)} = 0.654$	ns
Residual	0.0751	12	0.0063		

Table S2. Results of the post hoc Bonferroni's comparison test for differences in biovolume toxicity relative to STX analogues produced by *Raphidiopsis raciborskii* T3 exposed (infochemicals) and not exposed (control) to *Daphnia gessneri* over 6 days of incubation. ns = no significant differences. CI = confidence interval.

CONTROL—INFOCHEMICALS (biovolume toxicity)	Mean Difference	95%CI of Differences	*P*
**Time (d^{-1})**			
**0**	0	−0.1737–0.1737	ns
**2**	-0.03	−0.2037–0.1437	ns
**4**	-0.06667	−0.1071–0.2404	ns
**6**	-0.3233	−0.4971–0.1496	<0.001

Table S3. Results of the two-way ANOVA for differences in the relative expression of *sxtI* and *sxtU* by *Raphidiopsis raciborskii* T3 exposed (infochemicals) and not exposed (control) to *Daphnia gessneri* over 6 days of incubation. **ns**= no significant differences.

sxtI	SS	Df	MS	*F*	*P*
Time	1.307	3	0.4356	$F_{(3,12)} = 8.172$	<0.01

Infochemicals	0.5646	1	0.5646	$F_{(1,4)} = 8.588$	<0.05
Interaction	1.621	3	0.5402	$F_{(3,12)} = 10.13$	<0.01
Subjects (matching)	0.263	4	0.0657	$F_{(4,12)} = 1.233$	ns
Residual	0.6396	12	0.0533		
sxtU	**SS**	**Df**	**MS**	***F***	***P***
Time	5.36	3	1.787	$F_{(3,12)} = 137.7$	<0.0001
Infochemicals	2.237	1	2.237	$F_{(1,4)} = 153.2$	<0.001
Interaction	3.335	3	1.112	$F_{(3,12)} = 85.65$	<0.0001
Subjects (matching)	0.0584	4	0.0146	$F_{(4,12)} = 1.125$	ns
Residual	0.1557	12	0.0130		

Table S4. Results of the two-way ANOVA for differences in the biovolume growth curves and chlorophyll-a of *Raphidiopsis raciborskii* T3 exposed (infochemicals) and not exposed (control) to *Daphnia gessneri* over 6 days of incubation. **ns=** no significant differences.

**Control**	**SS**	**Df**	**MS**	***F***	***P***
Time	0.2598	3	0.08661	$F_{(3,60)} = 165.9$	<0.0001
Infochemicals	0.5474	9	0.06082	$F_{(9,20)} = 107.5$	<0.0001
Interaction	0.05403	27	0.00200	$F_{(27,60)} = 3.833$	<0.0001
Subjects (matching)	0.01132	20	0.00056	$F_{(20,60)} = 1.084$	ns
Residual	0.03132	60	0.00052		
**Infochemicals**	**SS**	**Df**	**MS**	***F***	***P***
Time	0.2608	3	0.08695	$F_{(3,60)} = 920.9$	<0.0001
Infochemicals	0.6307	9	0.07008	$F_{(9,20)} = 627$	<0.0001
Interaction	0.07168	27	0.00265	$F_{(27,60)} = 28.12$	<0.0001
Subjects (matching)	0.00223	20	0.00011	$F_{(20,60)} = 1.184$	ns
Residual	0.0057	60	0.00009		

Table S5. Efficiencies and standard curve parameters obtained by real-time qPCR analysis for the cyanobacterial *rpoC1-*, *sxtI-* and *sxtU*-specific primer sets.

**Target Gene**	**Strain**	**Efficiency**	**Slope**	***Y*-intercept**	R^2	**Melting Temperature**
**Cyanobacterial *rpo*C1**	*R. Raciborskii* T3 genomic DNA	98.23	−3.365	13.49	0.9794	60–95 °C

**Cyanobacterial *sxt*I**	*R. Raciborskii* T3 genomic DNA	107.18	−3.161	16.27	1.0000	60–95 °C
**Cyanobacterial *sxt*U**	*R. Raciborskii* T3 genomic DNA	107.52	−3.154	15.43	0.9997	60–95 °C

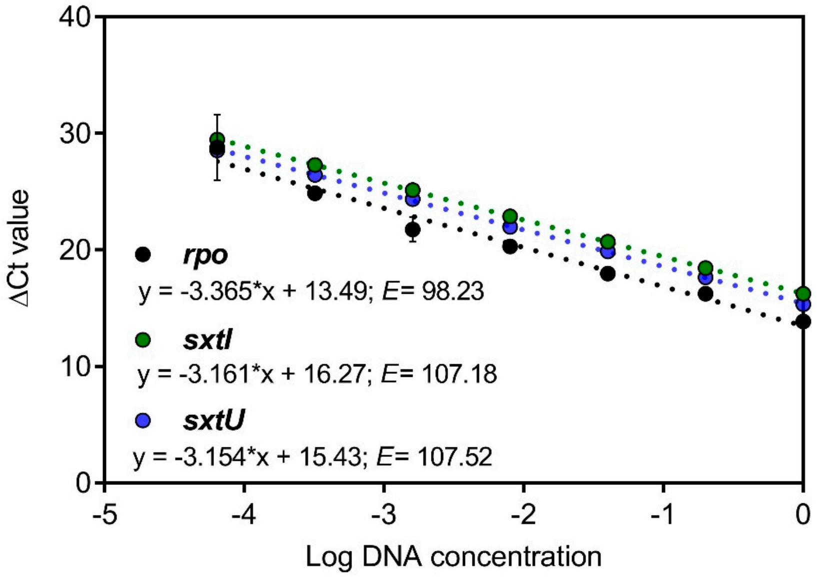

Figure S1. Standard curve and its linear equation for cyanobacterial *rpoC1-*, *sxtI-* and *sxtU*-specific primers.
